# Global burden of ischemic heart disease in older adult populations linked to non-optimal temperatures: past (1990–2021) and future (2022–2050) analysis

**DOI:** 10.3389/fpubh.2025.1548215

**Published:** 2025-02-12

**Authors:** Lihui Liu, Yisong He, Gang Huang, Yangxi Zeng, Jiaan Lu, Ru He, Haiqing Chen, Yuheng Gu, Qingwen Hu, Bin Liao, Juyi Wan

**Affiliations:** ^1^Department of Cardiovascular Surgery, The Affiliated Hospital, Southwest Medical University Metabolic Vascular Diseases Key Laboratory of Sichuan Province, Key Laboratory of Cardiovascular Remodeling and Dysfunction, Luzhou, China; ^2^Clinical Medical College, Southwest Medical University, Luzhou, China

**Keywords:** ischemic heart disease, global burden of disease, older adult population, non-optimal temperatures, estimated annual percentage change

## Abstract

**Background:**

Ischemic heart disease (IHD) is a leading cause of death and disability, particularly affecting the older adult population. Extreme temperatures, especially very low and very high temperatures, are known to exacerbate cardiovascular disease burden. With the ongoing global climate change, understanding the impact of non-optimal temperatures on IHD burden becomes increasingly important, especially in vulnerable populations such as the older adult.

**Methods:**

This study used data from the Global Burden of Disease Study 2021 (GBD 2021) to analyze the spatiotemporal trends of low and high temperatures on IHD burden in the older adult population (aged 60 and above) from 1990 to 2021. We used age-standardized rates (ASR), annual percentage change (EAPC), and the Bayesian age-period-cohort (BAPC) model to forecast 2050. Additionally, the geographic differences in IHD burden were analyzed using World Bank regions.

**Results:**

From 1990 to 2021, the IHD burden in the older adult population was mainly attributed to low temperatures. However, it has increased the burden of IHD due to high temperatures, especially in tropical and low-income regions. The analysis of gender difference revealed that men are usually more affected by high temperatures, though generally, women are more sensitive to low temperatures. Forecasts are that in the future, the burden of IHD due to high temperatures will continue to rise, especially in areas with limited adaptive capacity.

**Conclusion:**

Although low temperature remains the most important contributor to IHD burden among the older adult, the burden attributable to high temperature is on the rise, which increases the need to address the extreme temperature fluctuation. That is more so in poor-income and tropical regions where the most vulnerable populations bear a higher risk for health. Thus, there is an urgent need to develop adaptive public health measures against the dual health risks from extreme temperatures. The findings emphasize that targeted interventions are necessary, with adjustments in regional differences and gender-specific risks to effectively address the growing health threats from climate change.

## 1 Introduction

Ischemic heart disease refers to a category of heart disease wherein the coronary arteries do not get an adequate amount of blood supply and is very closely associated with a pathological process such as atherosclerosis, thrombosis, and myocardial ischemia (1). These processes contribute to the narrowing of the coronary arteries, which impairs blood flow and exacerbates the risk of ischemic events (2). IHD is the leading cause of death in many parts of the world and accounts for much destruction to health and quality of life throughout the world. According to the 2021 study on the Global Burden of Disease, IHD remains one of the leading causes of mortality worldwide, with a great burden in low-income and middle-income countries (3). The main clinical manifestations of IHD are angina, myocardial infarction, and heart failure. In line with aggravation of the disease, there is a growth of risks for patients as far as adverse cardiovascular events, such as myocardial infarction, development of congestive heart failure, and sudden death. A study has shown that patients with IHD have highly significant deterioration in quality of life and huge medical resources used by the patients, hence making burdens larger on an individual and on society. In recent years, the accelerated process of aging worldwide, especially the increasing proportion of people aged 60 and older, has contributed to an increased health burden from IHD. Whereas in 1990 the population aged 60-plus accounted for 9.2% of the total global population, this percentage increased to 11.7% in 2013 and is projected to reach 21.1% by 2050 (4). As the population ages, the incidence of cardiovascular diseases rises sharply, and the burden of IHD is more pronounced among the older adult. (5). The burden of IHD is closely linked to traditional risk factors such as hypertension, diabetes, smoking, and physical inactivity. However, abnormal temperature exposure has emerged as a new health challenge, significantly impacting the global burden of IHD (6).

With the rising climatic changes due to greenhouse gas emission, the current interest in public health has gained high interest lately. The abnormal rise of the ambient temperature has shown to bring an immense effect on the life of a human species. According to the findings presented in “Lancet Countdown on Climate Change, it is seen as a huge threat to the health consequences to make 21st-century history” (7). Since 1981, the global annual average temperature has risen about 0.18°C per decade (8). The 2019 global land and sea surface temperature was 0.95°C above the historical average and the second hottest on record (9). Temperature extremes-heatwaves and cold spells-linked to climate change are some of the current major global public health issues (10). Abnormal temperature is generally regarded as a major risk factor for cardiovascular disease, and epidemiological evidence indicates clearly that the extremes of high and low temperatures are strongly associated with cause-specific mortality and morbidity. High temperature can increase the heart workload due to increased blood circulation, heart rate, and blood pressure, which may trigger acute myocardial infarction and other events related to IHD (11). Conversely, low temperatures cause vasoconstriction, increase blood viscosity, and promote thrombosis, further increasing the risk of IHD (12). As extreme temperature events caused by climate change occur more frequently, the global burden of ischemic heart disease is expected to intensify, particularly under the alternating influence of high and low temperatures, with vulnerable populations (such as the older adult and high-risk patients) facing greater health risks (13).

This study aims to explore the impact of abnormal temperatures on the global burden of ischemic heart disease (IHD) from 1990 to 2021, with a particular focus on gender-specific trends in the older adult population. Based on data from GBD, the regional IHD mortality rates and DALY have been analyzed, its etiological characteristics, and related gender-specific differences both at the global level and based on different regions (of classification by the World Bank). Additionally, we will predict the trend of IHD burden from 2022 to 2050, focusing on the impact of abnormal temperatures on older adult populations, female groups, and high-risk populations.

## 2 Methods

### 2.1 Data sources

This study makes use of data from the Global Burden of Disease 2021, estimating the burden of IHD amongst the older adult population (aged above 60 years) by gender, with non-optimal temperatures (both high and low) attributable to it (14). The data relates to mortality rates, DALYs, and related risk factors specifically for temperature exposure categories: high temperature, low temperature, and suboptimal temperature. The analysis covers the period from 1990 to 2021 and has been stratified by income level using the World Bank’s income classification, dividing countries into low, middle, and high-income regions, rather than the regional classification used by the Global Burden of Disease (GBD) study. This approach allows for an examination of the differences in IHD burden across these income groups under extreme temperature conditions.

### 2.2 Data cleaning and filtering

The data was then filtered to include only relevant measures, namely DALYs and rates due to temperature exposure, which were further filtered into high temperature, low temperature, and suboptimal temperature. Key variables selected for analysis included: measure (DALYs), sex (both sexes), metric (rate), risk factor (temperature exposure under high, low, and suboptimal conditions), and time period (1990–2021).

To enhance clarity, the temperature exposure categories were renamed as follows (15): “High temperature” → “High Temp,” “Low temperature” → “Low Temp,” “Suboptimal temperature” → “Non-optimal Temp.”

Furthermore, a new variable labeled direction was introduced to represent the trend of estimated annual percentage change (EAPC), indicating whether the DALY values showed a positive or negative trajectory over time.

### 2.3 Estimated annual percentage change (EAPC)

Trend estimates in DALYs from heat and cold exposure for geographic units have been measured by the estimated annual percentage change (EAPC) between 1990 and 2021. The EAPC is defined as the average annual percentage rate of the trend or annual rate of change, representing the degree of changing trends over time when given ASRs (16–18).

The EAPC was calculated using the following formula:

$$\mathrm{EAPC}\;=\;100\times \left(e^{\beta_{1}}-1\right)$$

Here: $\beta_{1}$ is the slope from the linear regression of the logarithmic transformation of age-standardized rates (ASR).

Lower and upper values of the EAPC were also considered for the estimation of uncertainty intervals (95% UI).

A linear regression model was used to estimate the EAPC (18–20):

$$\mathrm{In}\left(ASR\right)\;=\;\beta_{0}\;+\;\beta_{1}\;x+\;\in$$

Here: $\ln\left(ASR\right)$ represents the natural logarithm of the age-standardized rates (ASMR or ASDR). x represents the calendar year (1990, 1991, …, 2021). $\beta_{0}$ is the intercept, and $\beta_{1}$ represents the annual change in ASR. ∈ is the error term.

### 2.4 Future projections (2022 to 2050)

Beyond historical analysis, this study projected the future burden of IHD attributable to non-optimal temperatures among older adult populations. To project future trends in ASMR and ASDR rates under high- and low-temperature conditions up to 2050, the Bayesian Age-Period-Cohort (BAPC) model was fitted (21, 22).

We used the BAPC model for its flexibility in dealing with age, period, and cohort effects, which is especially suitable for the long-term forecast of the impacts of extreme temperatures on IHD burden. Compared with the traditional regression model, the BAPC model fitted the nonlinear trend and complex temporal dynamic better. It is thus an ideal tool to analyze the evolving burden of IHD under global warming and the increased frequency of extreme temperature events (23).

### 2.5 Statistical analysis

The analyses were performed in R software, version 4.3.2, one of the most used tools for statistical computing and data visualization. Key packages employed in this analysis to ease the process of manipulation and visualization of data include: ggplot2: used for the development of high-quality visualizations for the better presentation of data. Dplyr: used for efficient cleaning and transformation of data. Readr: used for reading and importing structured data files into the R environment.

Together, these tools put together allowed for an organized, reproducible workflow that yields robust and accurate analytical outputs.

## 3 Results

### 3.1 Global burden of ischemic heart disease (IHD) due to suboptimal temperatures (1990–2021)

**Figure 1:**
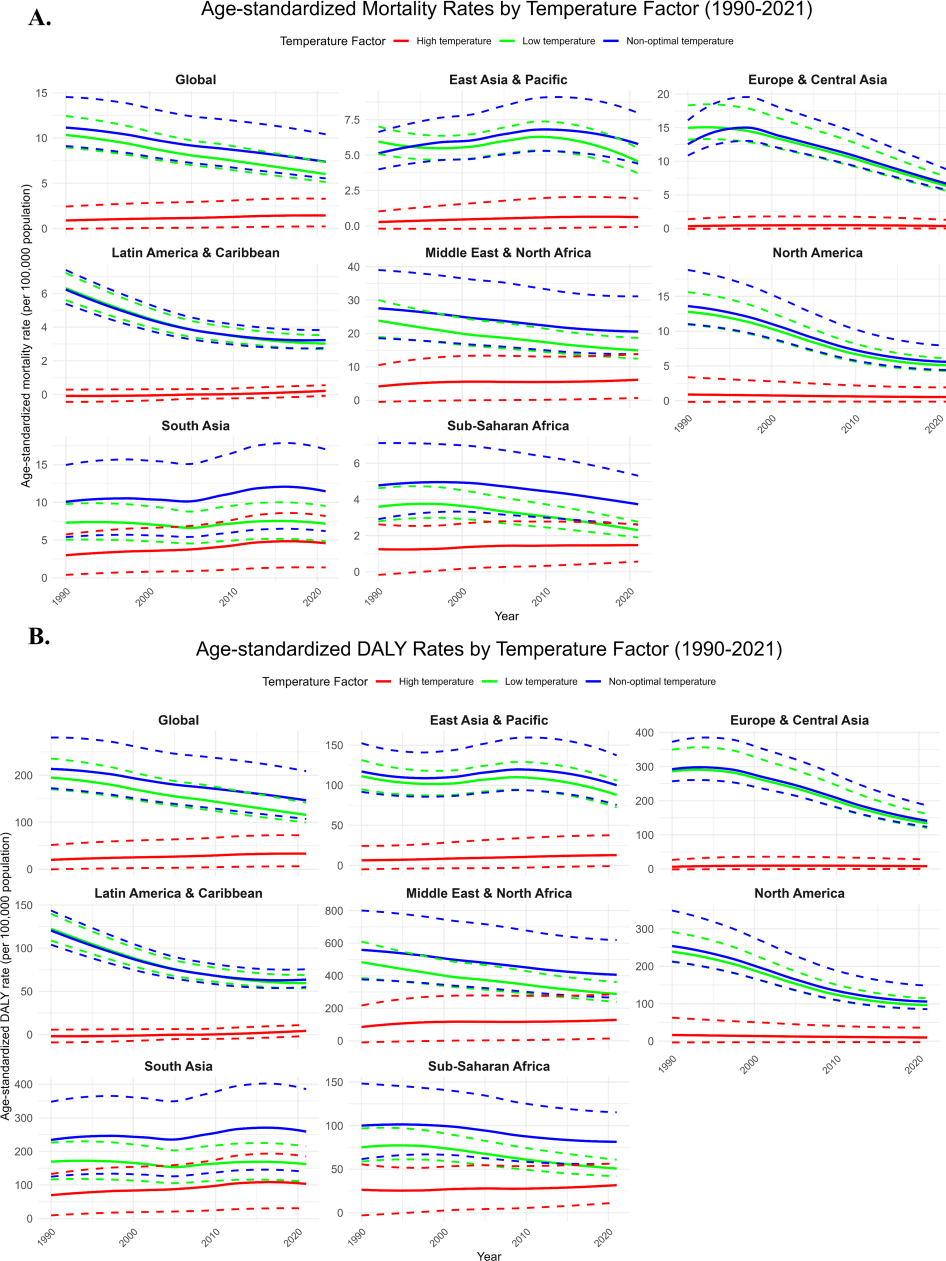
Temporal trends. Due to the impact of low, high, and non-optimal temperatures on ischemic heart disease in the older adult population, the age-standardized mortality rate (per 100,000 population) **(A)** and age-standardized disability-adjusted life years (DALYs) (per 100,000 population) **(B)** globally and in seven World Bank regions, from 1990 to 2021. DALYs, disability-adjusted life years. The dashed area represents the 95% uncertainty intervals.

**Figure 2:**
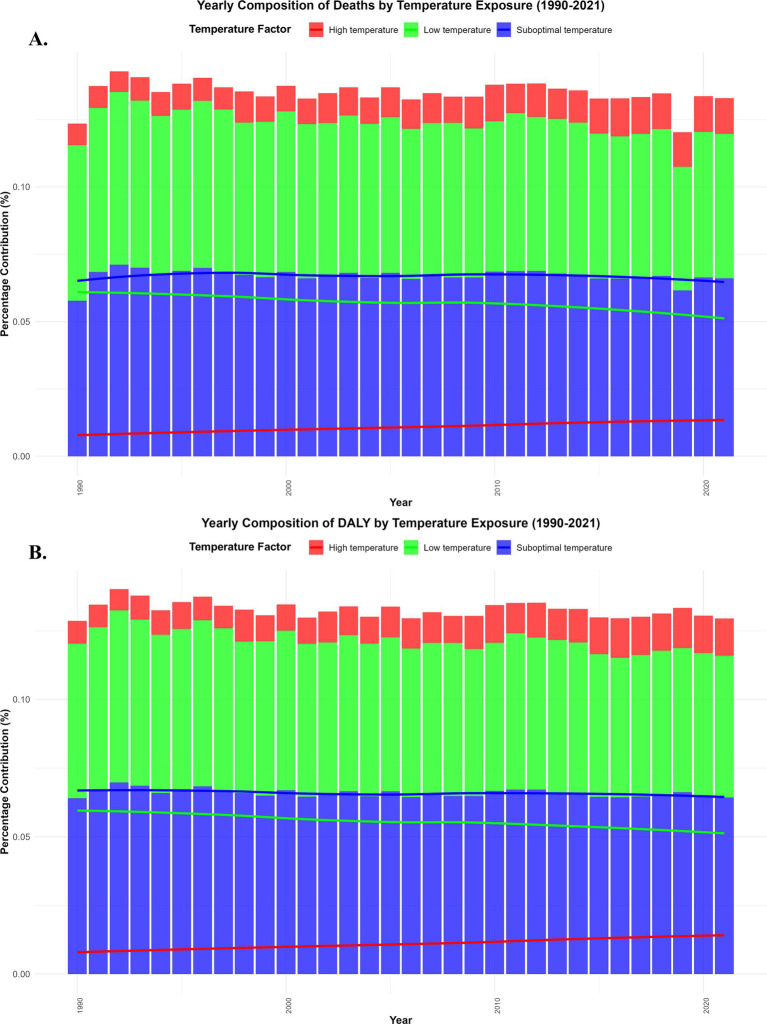
Global trends in IHD burden due to low and high temperatures (1990–2021). Annual percentage of IHD deaths **(A)**. Annual percentage of disability-adjusted life years (DALYs) **(B)**.

During 1990–2021, the global burden of IHD related to low temperature was significantly higher than that related to high temperature (Figure 1). The IHD mortality rate related to low temperatures showed a declining trend, with an ASMR that decreased from 9.74 (95% UI: 8.52, 11.60) in 1990 to 6.14 in 2021, with an EAPC of −0.37%. That means the low-temperature effects on IHD have, to a great extent, reduced over the years globally but with greater improvement in the high-income nations. This will be in line with improved medical technologies and better adaptation to climate factors in these areas (Figures 1, 2).

By contrast, the IHD mortality rate due to high temperature increased uninterruptedly from 0.90 in 1990 to 1.34 in 2021, with an EAPC of 0.48%. This indicates that over time, high temperatures have increasingly contributed to the global burden of IHD. The health effect of high temperatures on the older adult has been exacerbated by increasing frequency of extreme climate events, especially in low-income and middle-income countries (Figures 1, 2).

**Figure 3:**
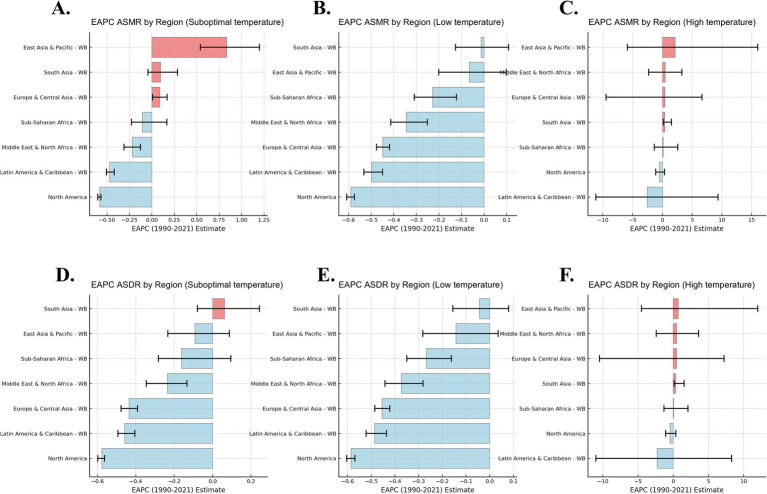
Annual Percentage Change (EAPC) of IHD Mortality and DALYs Attributable to Low and High Temperatures (1990–2021). This figure presents the EAPC of Age-Standardized Mortality Rates (ASMR) and Age-Standardized Disability-Adjusted Life Years (ASDR) for suboptimal **(A, D)**, low **(B, E)**, and high **(C, F)** temperature exposure across World Bank regions from 1990 to 2021.

The contribution of low and high temperatures to the IHD burden showed significant annual fluctuations from 1990 to 2021. Low temperature exposure had a relatively large impact on both mortality and disability-adjusted life years (DALYs), particularly in colder regions, where the cardiovascular health of older adult populations was significantly affected. In contrast, the impact of high temperature exposure on mortality and DALYs increased year by year, particularly in tropical and subtropical regions. As global temperatures rise, the IHD burden caused by high temperature exposure continues to worsen (Figures 2, 3). The percentage of IHD deaths (Figure 2A) and DALYs (Figure 2B) attributable to low temperatures exhibited a fluctuating trend globally, but the overall trend was a decline, particularly in high-income countries. This was due to improvements in medical conditions and enhanced climate adaptation capabilities, which led to a reduction in the burden of low temperature exposure over the years. On the other hand, the burden of IHD caused by high temperatures has been increasing year by year, especially in tropical regions such as Sub-Saharan Africa and Southeast Asia, where the frequency of extreme high-temperature events has increased, making the health risks of heatwaves even more severe (Figures 2, 3).

Based on the EAPC analysis from 1990 to 2021, the burden of IHD related to low temperatures has shown negative growth globally, particularly in high-income regions, where, with improved climate adaptation, the IHD mortality rate and DALYs related to low temperatures have declined each year. In contrast, the IHD mortality rate and DALYs associated with high temperatures in low- and middle-income countries showed positive EAPC values, indicating that as global temperatures rise, the burden of IHD on older adult populations in these regions is increasing year by year (Figure 3). The impact of low temperatures has decreased annually, especially in high-income countries, benefiting from improvements in health infrastructure and climate adaptation. However, the impact of high temperatures has shown a continuous upward trend, especially in low- and middle-income countries, where older adult populations face higher health risks, particularly in tropical and subtropical regions like Southeast Asia and Sub-Saharan Africa (Figures 1–3).

### 3.2 Regional burden of ischemic heart disease (IHD) due to suboptimal temperatures by World Bank region (2021)

**Figure 4:**
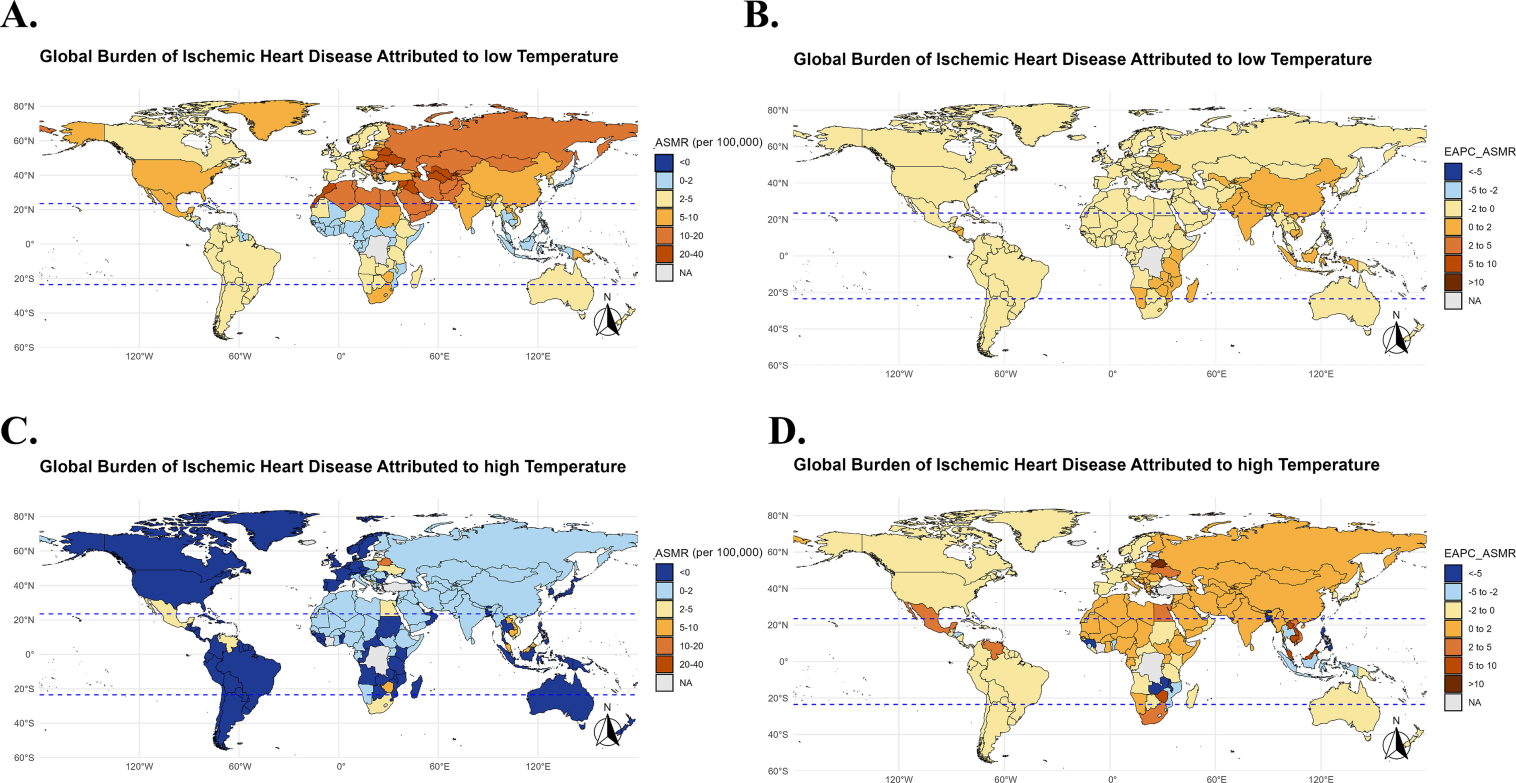
Global burden of IHD due to low temperatures in 2021 and EAPC analysis (1990–2021). Global map showing the IHD mortality rate (ASMR) due to low and high temperatures in 2021 **(A,C)**, Global map of the annual percentage change (EAPC) in IHD mortality rates due to low and high temperatures from 1990 to 2021 **(B,D)**.

**Figure 5:**
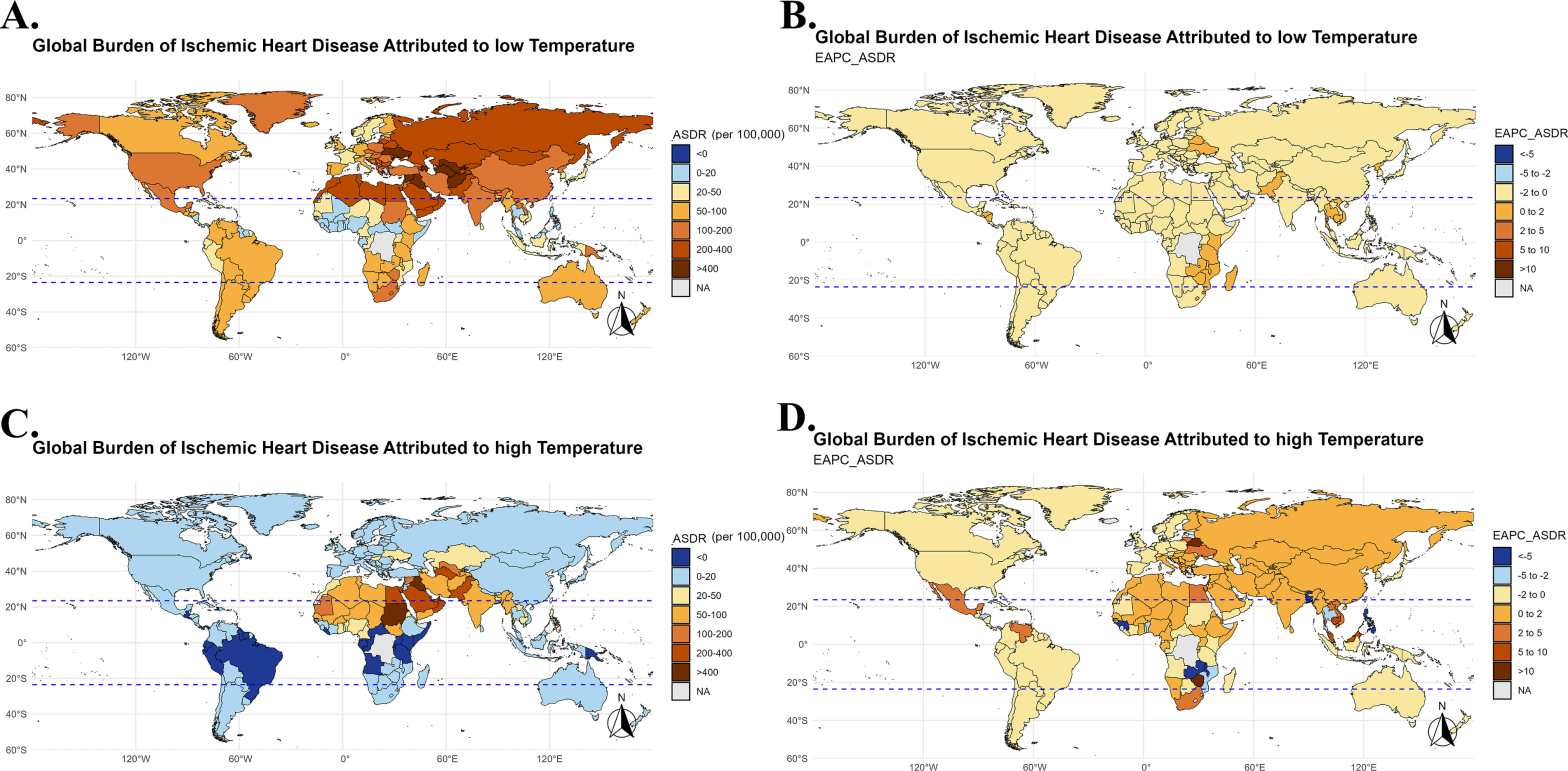
Global burden of IHD due to low temperatures in terms of DALYs in 2021 and EAPC analysis (1990–2021). Global map of IHD disability-adjusted life years (DALYs) due to low and high temperatures in 2021 **(A,C)**, Global map of the annual percentage change (EAPC) in IHD DALYs due to low temperatures from 1990 to 2021 **(B,D)**.

**Figure 6:**
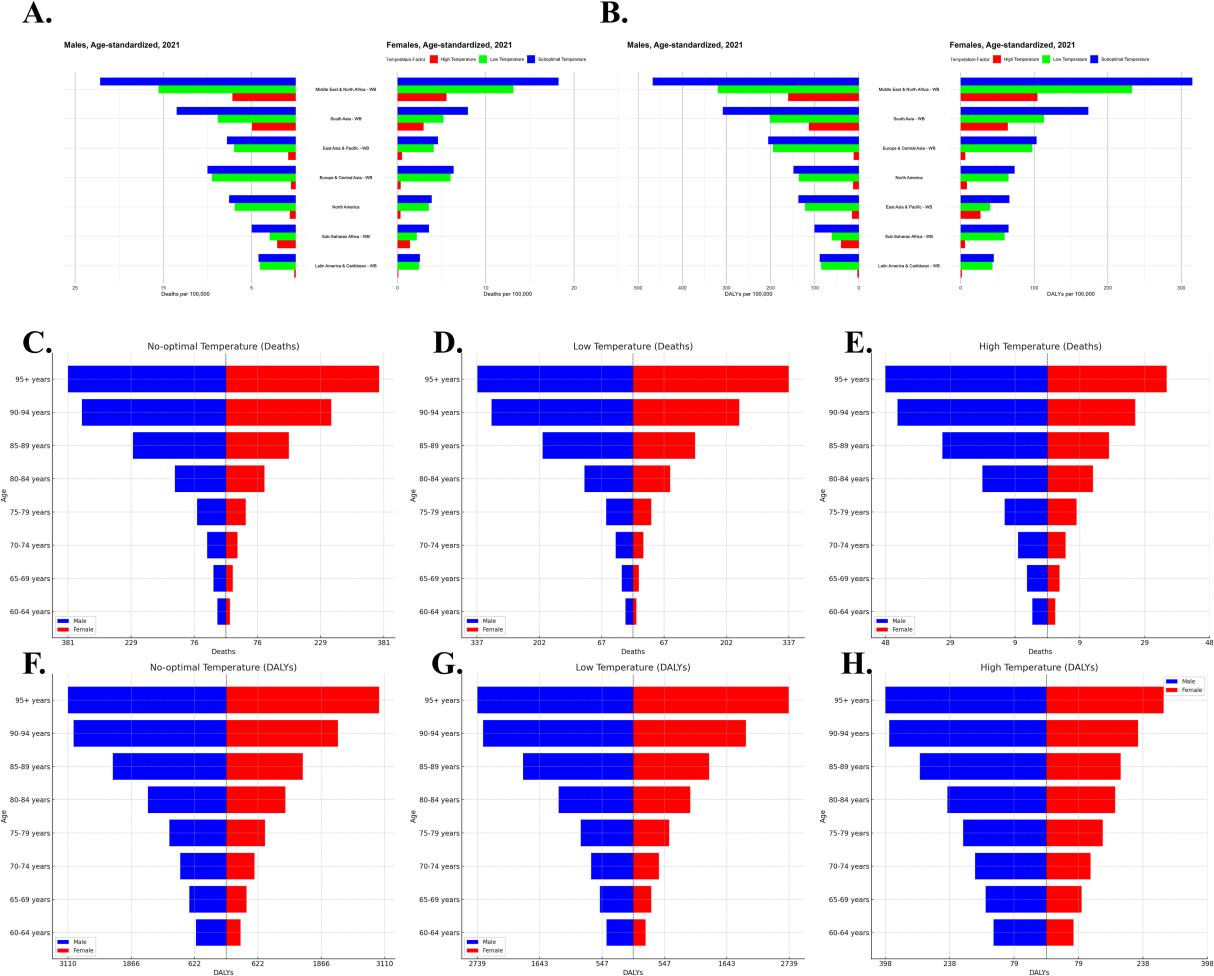
Gender-specific global burden of IHD in 2021 due to suboptimal temperatures. Age-standardized IHD mortality and disability-adjusted life years (DALYs) across seven World Bank regions for males **(A)** and females **(B)** under suboptimal temperatures (low and high). IHD deaths by age group for males (blue) and females (red) under no-optimal temperature **(C)**, low-temperature exposure **(D)** and high-temperature exposure**(E)**. IHD DALYs by age group under no-optimal temperature **(F)**, low-temperature exposure **(G)** and high-temperature exposure **(H)** for males (blue) and females (red).

In 2021, the burden of IHD related to low temperatures remained high in certain regions, particularly in Eastern Europe, Sub-Saharan Africa, South Asia, and Central Europe. In these regions, the IHD mortality rate and disability-adjusted life years (DALYs) caused by low temperatures were relatively high, especially in countries such as Bulgaria, Lesotho, Afghanistan, and Estonia, where the older adult population was significantly affected (Figures 4A, 5A, 6A). At the same time, in the North Africa and Middle East regions, the burden of IHD related to high temperatures was more prominent, particularly in countries such as the United Arab Emirates, Saudi Arabia, and Mauritania. As temperatures rise, the burden of IHD in these regions has significantly increased (Figures 4B, 5B, 6A).

In high-income regions (such as Europe and North America), the burden of IHD due to low temperatures has been decreasing over the years, while the burden of IHD related to high temperatures has significantly increased in low-income regions (such as Sub-Saharan Africa and Southeast Asia). The burden of IHD due to low temperature exposure is declining in high-income countries, while the burden from high temperature exposure continues to rise in low-income countries, indicating that climate change has a particularly significant health impact in these regions (Figure 3).

### 3.3 IHD burden in 204 countries worldwide (1990–2021)

In 2021, the burden of IHD related to low temperatures was high in certain countries, particularly in Eastern Europe, Sub-Saharan Africa, South Asia, and Central Europe. Countries like Bulgaria, Lesotho, Afghanistan, and Estonia saw significant cardiovascular burdens in their older adult populations due to exposure to low temperatures (Figures 4, 5).

At the same time, in North Africa and the Middle East, the burden of IHD caused by high temperatures was also quite severe among the older adult population, especially in countries like the United Arab Emirates, Saudi Arabia, and Mauritania. As global temperatures rise, the cardiovascular health burden in these regions has sharply increased (Figures 4, 5). Particularly in low-income and tropical regions, the impact of extreme temperatures on IHD burden is more pronounced due to limited healthcare resources and adaptive capacity. These regions face greater challenges in mitigating the effects of temperature extremes, leading to an amplified health burden on the older adult population.

### 3.4 Gender and age differences analysis

According to the analysis, the male population has a higher burden of IHD under high-temperature exposure, particularly in low-income countries and tropical regions such as Southeast Asia and South Asia, where males face higher IHD mortality rates. On the other hand, the female population experiences a more significant burden under low-temperature exposure, especially in cold regions like Eastern Europe and Russia, where IHD mortality rates and Disability-Adjusted Life Years (DALY) increases are higher for women (Figures 6A,B). The burden of IHD for older adult individuals aged 80 and above increases significantly under both low and high-temperature exposures, especially among women. The burden on the male population is higher under low-temperature exposure, while the burden under high-temperature exposure, though slightly lower than that for women, still shows a consistent upward trend (Figures 6A,B).

### 3.5 Gender-specific projections of IHD burden in the older adult due to low and high temperatures from 2022 to 2050

**Figure 7:**
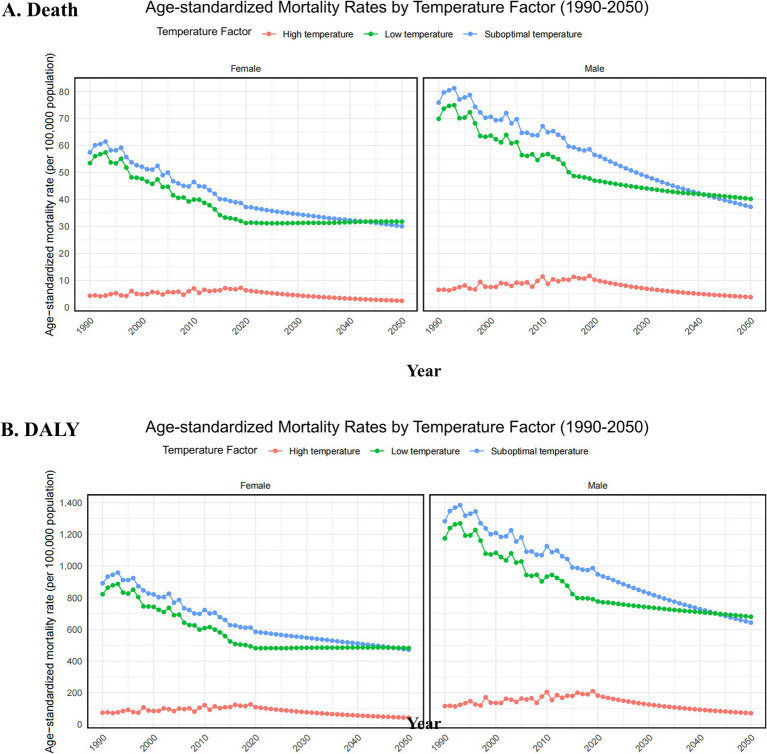
Projected sex-specific trends in age-standardized mortality rate (per 100,000 population) **(A)** and age-standardized disability-adjusted life years (DALYs) rate (per 100,000 population). **(B)** For the older adult population, attributable to low, high, and non-optimal temperatures causing ischemic heart disease, from 2022 to 2050. DALYs, disability-adjusted life years.

According to the forecast analysis, from 2022 to 2050, the burden of ischemic heart disease (IHD) in the global older adult population due to suboptimal temperatures (both high and low) is expected to show a continuous upward trend (Figure 7). Specifically: Female older adult population: Under low temperature exposure, the IHD burden is expected to continue decreasing. However, under high temperature exposure, both the IHD mortality rate and disability-adjusted life years (DALYs) are projected to gradually increase. In particular, in the coming decades, the global rise in temperatures is likely to exacerbate this effect, leading to a significant increase in the health burden for the female population. Male older adult population: In contrast to females, the male older adult population is projected to experience a decreasing IHD burden under both high and low temperature exposure. Although there are some fluctuations in the forecast for future changes, overall, the IHD burden in males does not seem to rise as sharply as it does in females with high temperature exposure.

## 4 Discussion

In this study, we conducted a detailed analysis of the impact of non-optimal temperatures (both low and high) on the burden of ischemic heart disease (IHD) globally from 1990 to 2021. Our results indicate that the burden of IHD due to low temperatures has shown a declining trend, particularly in high-income countries, primarily due to advancements in medical technology and enhanced climate adaptation capabilities. However, the burden of IHD caused by high-temperature exposure has shown a continuous upward trend worldwide, especially in low- and middle-income countries. Climate change has intensified the frequency and severity of heatwaves, posing an increasingly severe health threat, especially to the older adult population (24).

Particularly, in countries with high incomes, the IHD burden due to low temperatures has been reduced, while health risks from high-temperature exposure have increased incrementally (25). This has been linked with improved health infrastructure and better adaptation to climatic changes in such regions. However, in the low-income and middle-income countries, this is not so. In the context of global rising temperatures, the burden of IHD due to high-temperature exposure has been growing year by year, especially in tropical and subtropical areas like sub-Saharan Africa and Southeastern Asia, where extreme heat events have become an emerging health threat (26).

It is also important to notice the significant role played by gender and age differences on the IHD burden. While the burden in the male population is higher under exposure to high temperature, females experience more significant burdens under low-temperature exposure. This can be attributed to biological differences such as fat distribution and hormone levels (27). Specifically, women are less adaptable to low temperatures, while men experience a higher cardiovascular burden in high-temperature environments due to typically higher heart rates and blood pressure. Particularly in Eastern Europe, Russia, and other cold regions, the IHD mortality rate and disability-adjusted life years (DALYs) in female populations under low-temperature exposure have increased more significantly. For the older adult population, especially those aged 80 and above, the burden of IHD due to both low and high-temperature exposure is even more severe, presenting a major public health challenge for the global older adult population.

According to future projections, from 2022 to 2050, the IHD burden caused by non-optimal temperatures in the older adult population is expected to continue increasing, especially in female older adult populations. It is anticipated that high-temperature exposure will further exacerbate the IHD health burden. This trend emphasizes the long-term impact of global temperature rise and the important role of gender and age in health risks. However, while the BAPC model is suitable for projecting long-term impacts, under extreme climate change scenarios, the model’s assumptions may affect the accuracy of the projections, mainly in terms of the incompleteness of data for some low-income countries (28).

Therefore, to mitigate the IHD burden, especially in low- and middle-income countries, there is a global need to strengthen climate adaptation efforts, improve public health policies, and implement intervention measures targeting the older adult population. The extreme temperature-related public health strategy in low-income regions should involve providing cooling facilities, health monitoring systems, and early warning systems. Furthermore, enhancement of basic healthcare infrastructure and better health management of older adult populations would help reduce the impact of extreme temperature on IHD burden. Besides, personalized health response strategies are needed to cope with changes in different climate zones and population characteristics toward a healthy society under climate change.

## 5 Conclusion

This study describes the global burden of IHD due to non-optimal temperature, both low and high, from 1990 to 2021, describing the disparate impacts of low and high temperatures on cardiovascular health. Low-temperature-related IHD has shown a declining trend worldwide since 1990, especially in high-income countries, where the adverse health effects of low-temperature exposure have gradually dropped with improvement in climate adaptation capacity and medical technology. However, the burden of IHD due to high-temperature exposure has continued to increase throughout the world, especially in the low- and middle-income countries where the increase in extreme climate events has exposed the older adult population to higher health risks.

Moreover, there is a clear difference in the burden of IHD from the perspective of gender and age. High-temperature exposure increases the burden among males, while in females, it increases during low-temperature exposure. The most severe burdens under both low- and high-temperature exposures were seen in the aged, particularly aged 80-plus years, thus reflecting a long-term health consequence of global climate change in the older adult population.

With the continuous increase in global temperatures, it is estimated that by 2050, the burden of IHD will continue to increase, especially the burden caused by high-temperature exposure. In view of this challenge, targeted measures should be taken by the whole world to enhance climate adaptation and guarantee the health of the older adult. The most important role that low- and middle-income countries can play in this context is to frame policies on public health, infrastructure, and health interventions regarding the older adult that would minimize the risk to health due to climate change.

## Data Availability

The original contributions presented in the study are included in the article/Supplementary material, further inquiries can be directed to the corresponding authors.

## Supplementary material

SUPPLEMENTARY TABLE 1 Global and World Bank region-specific age-standardized burden of ischemic heart disease attributable to non-optimal temperatures in aging populations (raw data).

SUPPLEMENTARY TABLE 2 Burden of ischemic heart disease due to non-optimal temperatures in the global older adult population by gender and age group (2021 raw data).

SUPPLEMENTARY TABLE 3 EAPC of the Burden of ischemic heart disease attributable to non-optimal temperatures in aging populations by World Bank region (1990-2021 raw data).

SUPPLEMENTARY TABLE 4 Age-standardized burden of ischemic heart disease due to non-optimal temperatures in the aging population of global countries (2021 raw data).

SUPPLEMENTARY TABLE 5 EAPC of the burden of ischemic heart disease attributable to non-optimal temperatures in the aging populations of global countries (1990-2021 raw data).

SUPPLEMENTARY TABLE 6 Burden of ischemic heart disease due to non-optimal temperatures in global older adult population by gender and age group (1990-2021 raw data and 2022-2050 projections).
